# Is vaspin related to cardio-metabolic status and autonomic function in early stages of glucose intolerance and in metabolic syndrome?

**DOI:** 10.1186/s13098-016-0165-1

**Published:** 2016-07-26

**Authors:** Rumyana Dimova, Tsvetalina Tankova, Georgi Kirilov, Nevena Chakarova, Lilia Dakovska, Greta Grozeva

**Affiliations:** 1 Department of Diabetology, Clinical Center of Endocrinology, Medical University Sofia, 2, Zdrave Str., Sofia, 1431 Bulgaria; 2 Department of Radioimmunology Laboratory, Clinical Center of Endocrinology, Medical University Sofia, Sofia, Bulgaria

**Keywords:** Vaspin, Glucose tolerance, Metabolic syndrome, Cardio-vascular autonomic function

## Abstract

**Background:**

This study aims to assess serum vaspin in early stages of glucose intolerance and in the presence of metabolic syndrome (MetS); and to evaluate vaspin correlation to different cardio-metabolic parameters and autonomic tone in these subjects.

**Methods:**

185 subjects (80 males and 105 females) of mean age 45.8 ± 11.6 years and mean BMI 31.2 ± 6.3 kg/m^2^, divided into groups according to: glucose tolerance, presence of MetS and cardio-vascular autonomic dysfunction (CAD), were enrolled. Glucose tolerance was studied during OGTT. Anthropometric indices, blood pressure, HbA1c, serum lipids, hsCRP, fasting immunoreactive insulin and serum vaspin were measured. Body composition was estimated by impedance analysis. AGEs were assessed by skin fluorescence. CAD was assessed by ANX-3.0.

**Results:**

There was no difference in vaspin levels between the groups according to glucose tolerance, presence of MetS, and CAD. Regression analysis revealed independent association between serum vaspin and total body fat in newly diagnosed type 2 diabetes (NDT2D) group, and between serum vaspin and age and total body fat in MetS group. Vaspin negatively correlated with both sympathetic and parasympathetic activity in normal glucose tolerance (NGT) and just with parasympathetic tone in NGT without MetS.

**Conclusion:**

Our results demonstrate no overt fluctuations in vaspin levels in the early stages of glucose intolerance and in MetS. Total body fat seems to be related to vaspin levels in MetS and NDT2D. Our data show negative correlation between vaspin and autonomic function in NGT, as vaspin is associated with parasympathetic activity even in the absence of MetS.

## Background

Vaspin is a visceral adipose tissue (VAT)-derived serine protease inhibitor with insulin-sensitizing effects. It is found in the VAT of Otsuka Long-Evans Tokushima Fatty rat, an animal model with central obesity and type 2 diabetes (T2D) [1]. Uncontrolled diabetes and weight reduction diminish vaspin expression, whereas the administration of insulin sensitizers, such as pioglitazone, normalizes its expression and serum concentration. In experimental conditions recombinant vaspin administration significantly improves glucose tolerance and insulin sensitivity [1, 2]. There is some evidence that vaspin has the potential to increase adipocyte insulin sensitivity and to suppress obesity through promoting the differentiation of 3T3-L1 preadipocytes by decreasing IL-6 mRNA and increasing GLUT-4 mRNA levels [3]. Based on these data it has been assumed that vaspin serves as an insulin sensitizer with anti-inflammatory effects [1, 2].

A meta-analysis, encompassing six studies including 1826 obese individuals and 11 studies including 1570 subjects with T2D has been conducted. It provides evidence of higher vaspin levels in obesity and T2D and emphasizes the pivotal role of vaspin in the progression of metabolic and glucose abnormalities [4].

The results of a growing number of studies in different ethnic groups have shown that cardiovascular autonomic dysfunction (CAD) is present in the early stages of abnormal glucose homeostasis [5, 6] and suggested its multifactorial modality and its relation to a number of cardio-metabolic risk factors. Central obesity is a confirmed risk marker for CAD [7], probably due to increased adipokine synthesis and secretion from VAT.

The impact of vaspin in the early stages of glucose intolerance is still poorly studied and the role of vaspin for the development of metabolic syndrome (MetS), diabetes and its chronic complications is not totally clarified. The present study aims to assess serum vaspin levels in the early stages of glucose tolerance impairments, in the presence of MetS, and CAD, and to evaluate the association between serum vaspin and different cardio-metabolic parameters and cardiovascular autonomic function (CAF) in these subjects.

## Methods

**Fig. 1:**
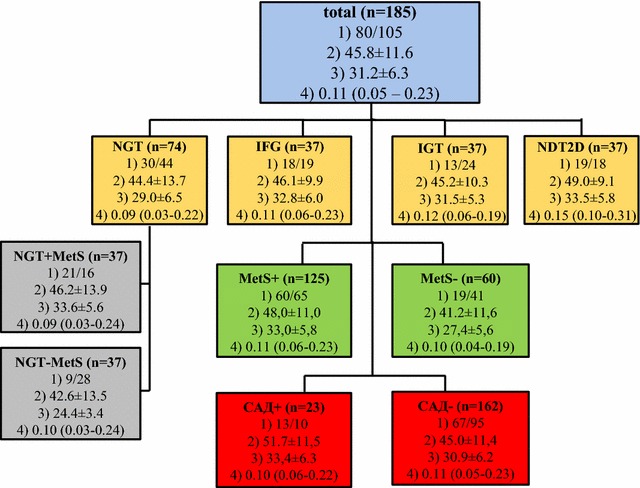
Main characteristics [number, gender distribution, mean age and body mass index (BMI)] of the participants in the groups according to glucose tolerance category—*NGT* normal glucose tolerance, *IFG* impaired fasting glucose, *IGT* impaired glucose tolerance, and newly-diagnosed type 2 diabetes *(NDT2D)*, with (MetS+) and without (MetS−) Metabolic syndrome, and with (CAD+) and without (CAD−) cardio-vascular autonomic dysfunction n—number, (1) gender (males/females), (2) age (years), (3) BMI (kg/m²), (4) serum vaspin concentration (ng/ml). Data is means ± SD and median (percentile 25–75 %)

A total of 185 subjects—80 males and 105 females, mean age 45.8 ± 11.6 (from 18 to 82 years), mean BMI 31.2 ± 6.3 kg/m^2^ were included in this cross-sectional study. They were divided into groups according to their glucose tolerance category, presence of MetS, and CAD. The main characteristics of the groups are displayed in Fig. 1.

Participants were recruited at the Department of Diabetology, Clinical Centre of Endocrinology, Medical University, Sofia within a screening program for diabetes mellitus.

All subjects declared their written informed consent and were familiar with the aims, methods and risks of participating in the study in accordance with the Helsinki Declaration and rules of Good Clinical Practice, as the study was approved by the Ethics Committee of the Medical University, Sofia.

All participants were interviewed for previously diagnosed diabetes and anti-diabetic therapy, arrhythmias and anti-arrhythmic drug administration, the presence of ischemic heart disease and experienced vascular events, which were adopted as exclusion criteria.

Anthropometric parameters were measured—height, weight and waist circumference (measured in a horizontal plane, the midline between the inferior margin of the 12th rib and the superior border of the iliac crest), and BMI was calculated.

Categories of glucose tolerance were evaluated during a standard oral glucose tolerance test with 75 g anhydrous glucose after an overnight fast. Fasting and a 120-min plasma glucose were examined by a hexokinase enzyme method (Roche Diagnostics). The glucose tolerance was defined according to 2006 WHO criteria. Fasting immunoreactive insulin (IRI) was measured using electrochemiluminescence immunoassay (ECLIA Roche Diagnostics) and homeostasis model assessment of insulin resistance and beta-cell function (HOMA-IR and HOMA-β) was defined according to Matthews et al. formulas [8].

Serum lipid parameters (total cholesterol, HDL cholesterol and triglycerides using an enzymatic colorimetric method (Roche Diagnostics), LDL cholesterol was calculated using Fridewald’s formula), HbA1c (NGSP certified) in whole blood samples using immunoturbidimetric method (Roche Diagnostics), high sensitive C-reactive protein (hsCRP) using a particle-enhanced turbidimetric method (CRP-Latex) (Roche Diagnostics), with an intra-assay coefficient of variation of less than 3.6 % and inter-assay coefficient of variation of less than 11.1 %, measuring range 0–160 mg/l (0–1600 mg/l with postdilution) and limit of quantification 0.085 mg/l, and serum vaspin using ELISA method (BioVendor), with an intra-assay coefficient of variation of less than 7.6 % and inter-assay coefficient of variation of less than 7.7 %, and limit of quantification 0.01 ng/ml, according to manufacturer᾽s protocol, were assessed in all participants at fasting.

Arterial blood pressure was measured in standard conditions. Body composition was estimated by impedance analysis (InBody 720). Visceral fat area was calculated in cm^2^ and total body fat was presented in  %.

The 2005 IDF definition of the MetS was used.

Tissue advanced glycation end products (AGEs) accumulation was assessed non-invasively measuring the skin autofluorescence of ultraviolet light on the ventral side of the lower arm (AGE-Reader-DiagnOpticsTM).

ANS function evaluation was performed with ANS-3.0 autonomic monitoring system (ANSAR Medical Technologies, Inc., Philadelphia, PA)—software that computes sympathetic and parasympathetic nervous system activity using “frequency-domain” analysis at rest and applying standard clinical tests: 1. deep breathing; 2. valsalva maneuver; and 3. standing from a seated position. The ANS-3.0 method, focused on the low-frequency range of the spectrum fixed between 0.04 and 0.15 Hz, computes sympathetic (low frequency area—LFa) and parasympathetic (respiratory frequency area—RFa) activity simultaneously and independently, applying spectral analysis of respiratory activity with concomitant spectral analysis of heart rate variability (HRV). The parasympathetic portion is defined as centered on the fundamental respiratory frequency-RFa and the remaining portion of the analysis interval of HRV spectrum corresponds to the sympathetic activity—LFa, measured in bpm^2^.

As 49 subjects presented with arterial hypertension (blood pressure >140/90 mmHg) on treatment with different classes of antihypertensive drugs, including beta- and alpha-blockers, the study was performed at least 24 h after the last dose of medications affecting autonomic function—antihypertensives, tricyclic antidepressants and SSRIs, refraining from coffee and smoking 12 h prior to the test, at least 30 min after the last meal, between 8 and 11am in the morning.

### Statistical analysis

Statistical analysis of the data was performed by SPSS 21.0 (SPSS, Chicago, USA). The data is expressed as mean ± standard deviation (SD) and median (percentile 25–75 %). Mann–Whitney U and Kruskal–Wallis one-way analysis of variance (One-way ANOVA) were used to compare independent non-normally distributed variables. Logarithmic transformation was used for skewed data distribution. Associations between normally distributed different measured parameters or their log scale were analyzed using Pearson correlation and stepwise multiple linear regression models. A p value (two tailed) of less than 0.05 was considered statistically significant.

## Results

**Fig. 2:**
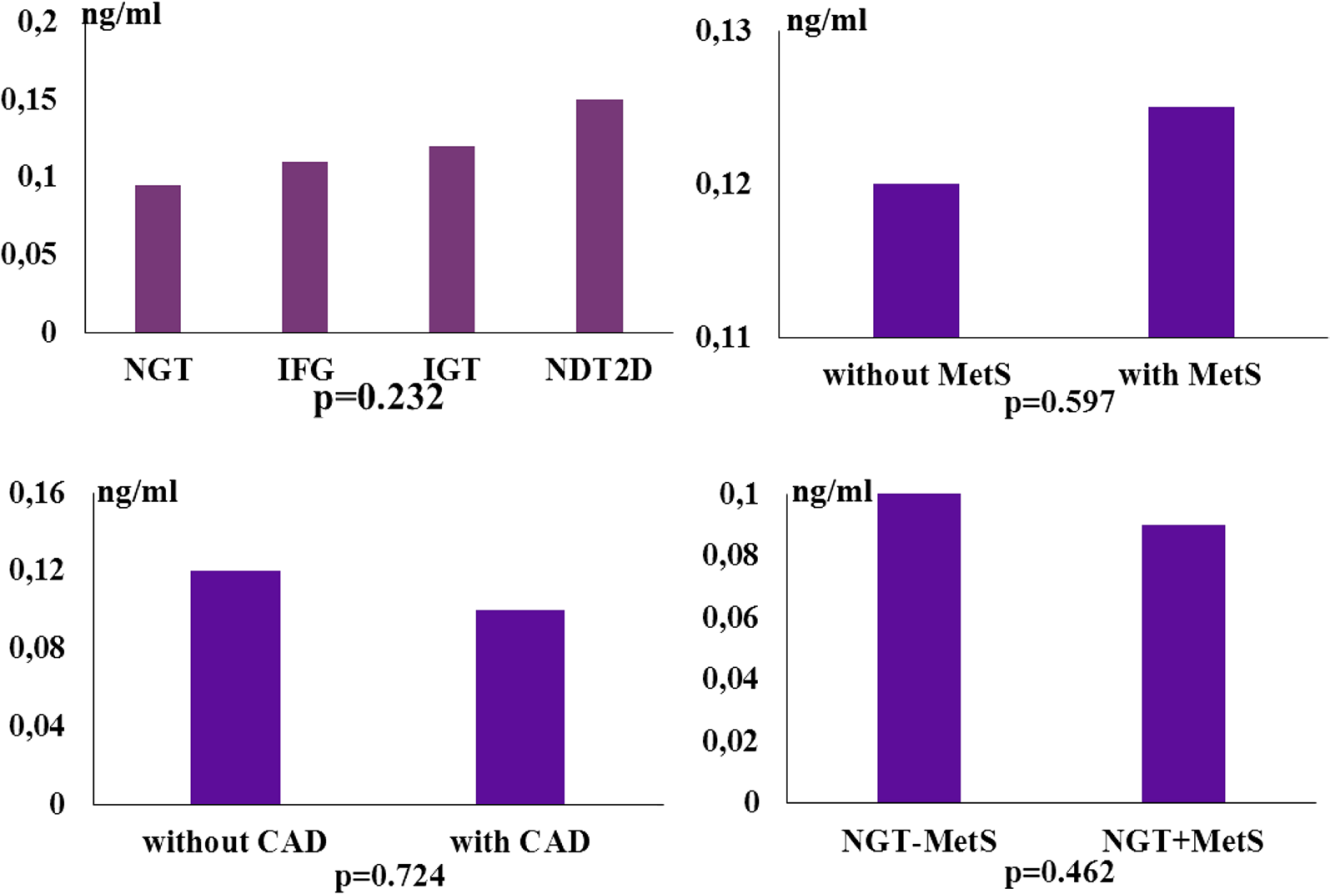
Serum vaspin levels in the groups according to glucose tolerance *NGT* normal glucose tolerance, *IFG* impaired fasting glucose, *IGT* impaired glucose tolerance, *NDT2D* newly-diagnosed type 2 diabetes, the presence of metabolic syndrome (without MetS and with MetS), the presence of cardio-vascular autonomic dysfunction (without CAD and with CAD), and in the NGT subgroups according to the presence of MetS (without NGT – MetS and with NGT + MetS)

No significant difference in serum vaspin levels between the groups according to glucose tolerance, presence of MetS, and CAD, as well as between the subgroups with normal glucose tolerance (NGT) with and without MetS was observed (Fig. 2). There were no gender differences in circulating vaspin levels (p = 0.078).

Serum vaspin correlated positively with total body fat and negatively with fasting plasma glucose and HbA1c in the newly-diagnosed type 2 diabetes group (NDT2D). The NGT + MetS group showed a positive correlation between serum vaspin and age, and AGEs, whilst in NGT–MetS group serum vaspin significantly correlated with age. The groups with MetS demonstrated a positive correlation between vaspin and age, total body fat, and AGEs, whereas in those without MetS there was a correlation just between vaspin and age (Table 1).

**Table 1:** Correlation between serum vaspin levels and age, glycemia, body fat distribution, indices for insulin secretion and insulin resistance (HOMA-β, HOMA-IR) and hsCRP in the groups according to glucose tolerance [normal glucose tolerance (NGT), impaired fasting glucose (IFG), impaired glucose tolerance (IGT) and newly-diagnosed type 2 diabetes (NDT2D)] and the presence of Metabolic syndrome (MetS)

	Vaspin			Vaspin	
	Corr coeff (r)	P		Corr coeff (r)	P
NGT			IFG		
Age	*0.35*	*0.003*	Age	0.16	0.373
Fasting plasma glucose	0.1	0.394	Fasting plasma gucose	0.01	0.992
HbA1c	0.17	0.157	HbA1c	−0.17	0.325
AGEs	0.18	0.144	AGEs	−0.12	0.486
BMI	−0.01	0.987	BMI	0.14	0.431
Total body fat	0.23	0.055	Total body fat	−0.05	0.762
Visceral fat area	0.12	0.318	Visceral fat area	0.22	0.212
Fasting serum insulin	−0.16	0.178	Fasting serum insulin	0.15	0.407
HOMA-β	−0.18	0.143	HOMA-β	0.12	0.483
HOMA-IR	−0.14	0.237	HOMA-IR	0.16	0.371
hsCRP	0.08	0.518	hsCRP	−0.23	0.188
NGT + MetS			IGT		
Age	*0.31*	*0.042*	Age	0.23	0.201
Fasting plasma glucose	0.05	0.768	Fasting plasma glucose	0.13	0.451
HbA1c	0.25	0.140	HbA1c	0.18	0.313
AGEs	*0.45*	*0.006*	AGEs	0.07	0.710
BMI	0.11	0.533	BMI	−0.28	0.107
Total body fat	0.28	0.110	Total body fat	−0.3	0.088
Visceral fat area	0.1	0.560	Visceral fat area	−0.25	0.156
Fasting serum insulin	−0.25	0.148	Fasting serum insulin	−0.1	0.451
HOMA-β	−0.25	0.148	HOMA-β	−0.19	0.286
HOMA-IR	−0.24	0.165	HOMA-IR	−0.05	0.779
hsCRP	0.14	0.438	hsCRP	−0.17	0.325
NGT – MetS			NDT2D		
Age	*0.41*	*0.015*	Age	0.05	0.791
Fasting plasma glucose	0.24	0.168	Fasting plasma glucose	*−0.4*	*0.023*
HbA1c	0.11	0.529	HbA1c	*−0.37*	*0.036*
AGEs	−0.05	0.799	AGEs	−0.11	0.539
BMI	−0.05	0.771	BMI	0.26	0.138
Total body fat	0.21	0.222	Total body fat	*0.43*	*0.013*
Visceral fat area	0.28	0.102	Visceral body fat	0.06	0.740
Fasting serum insulin	−0.01	0.939	Fasting serum insulin	0.12	0.504
HOMA-β	−0.09	0.618	HOMA-β	0.29	0.101
HOMA-IR	0.02	0.918	HOMA-IR	−0.06	0.736
hsCRP	0.11	0.543	hsCRP	−0.04	0.835
MetS+			MetS−		
Age	*0.22*	*0.019*	Age	*0.36*	*0.006*
Fasting plasma glucose	0.16	0.079	Fasting plasma glucose	0.18	0.185
HbA1c	0.15	0.110	HbA1c	0.09	0.531
AGEs	*0.2*	*0.036*	AGEs	−0.16	0.242
BMI	0.17	0.074	BMI	−0.13	0.348
Total body fat	*0.2*	*0.032*	Total body fat	0.04	0.797
Visceral fat area	0.16	0.094	Visceral fat area	0.01	0.958
Fasting serum insulin	0.06	0.525	Fasting serum insulin	−0.14	0.325
HOMA-β	−0.01	0.886	HOMA-β	−0.21	0.130
HOMA-IR	0.09	0.347	HOMA-IR	−0.08	0.553
hsCRP	0.01	0.916	hsCRP	−0.07	0.625

Statistical significant values are presented in italics

Serum vaspin concentrations presented a negative correlation with both sympathetic and parasympathetic activity at rest, during deep breathing, Valsalva, and standing in NGT group. The subjects in NGT + MetS subgroup showed a strong negative correlation between serum vaspin and sympathetic tone at rest, both sympathetic and parasympathetic tone during deep breathing, and standing, whilst in NGT-MetS subgroup vaspin negatively correlated just with parasympathetic power at rest and during applied clinical tests (Table 2). No significant correlation between serum vaspin and autonomic tone in the groups with glucose intolerance was observed.

**Table 2:** Correlation between serum vaspin levels and sympathetic (LFa) and parasympathetic (RFa) activity at rest and during clinical tests in the NGT group and in the subgroups with (NGT + MetS) and without (NGT – MetS) Metabolic syndrome

NGT	Vaspin	
	Corr coeff (r)	P
LFa at rest	−0.31	*0.010*
RFa at rest	−0.3	*0.010*
LFa deep breathing	−0.24	*0.042*
RFa deep breathing	−0.42	*<0.001*
LFa valsalva maneuver	−0.24	*0.043*
RFa valsalva maneuver	−0.33	*0.006*
LFa standing	−0.31	*0.009*
RFa standing	−0.34	*0.004*
NGT + MetS		
LFa at rest	−0.39	*0.021*
RFa at rest	−0.31	0.066
LFa deep breathing	−0.41	*0.016*
RFa deep breathing	−0.35	*0.038*
LFa Valsalva maneuver	−0.18	0.311
RFa Valsalva maneuver	−0.24	0.158
LFa standing	−0.36	*0.035*
RFa standing	−0.34	*0.049*
NGT – MetS		
LFa at rest	−0.2	0.251
RFa at rest	−0.38	*0.025*
LFa deep breathing	−0.05	0.774
RFa deep breathing	−0.51	*0.002*
LFa valsalva maneuver	−0.29	0.096
RFa valsalva maneuver	−0.42	*0.013*
LFa standing	−0.27	0.118
RFa standing	−0.38	*0.023*

Statistical significant values are presented in italics

A stepwise multiple regression analysis was conducted to assess the predictive value of these variables for serum vaspin levels. Age and total body fat entered into the regression and were related to vaspin in the presence of MetS (F [2, 113] = 7.86 (2, 113), p = 0.001), total body fat was related to vaspin in NDT2D (F [1, 33] = 6.01, p < 0.020), parasympathetic tone during deep breathing was related to vaspin in NGT–MetS (F [1, 33] = 8.92, p < 0.005), and sympathetic tone at rest was related to vaspin in NGT + MetS (F [1, 33] = 5.36, p < 0.027). The multiple correlation coefficients were 0.107, 0.403, 0.461 and 0.374, respectively, showing that 12.2 % of the variance of vaspin levels might be accounted for by age and total body fat in MetS, 16.2 % by total body fat in NDT2D, 21.3 % by parasympathetic activity during in NGT – MetS, and 14.0 % by sympathetic activity at rest in NGT + MetS (Table 3).

**Table 3:** Main determinants of serum vaspin in the groups with metabolic syndrome (MetS +), newly-diagnosed type 2 diabetes (NDT2D), normal glucose tolerance (NGT), and in the NGT subgroups with (NGT + MetS) and without (NGT–MetS) MetS

Stepwise multiple regression	(t, p)	F (df)	p	R	R^2^
Predictor variable					
Invaspin (MetS+)					
Model 1: age	(3.20, p = 0.002)	10.22 (1.114)	*p = 0.002*	0.074	0.082
Model 2: age total body fat	(3.10, p = 0.002) (2.26, p = 0.026)	7.86 (2.113)	*p = 0.001*	0.107	0.122
lnvaspin (NDT2D)					
Model 1: total body fat	(2.45, p < 0.020)	6.01 (1.33)	*p = 0.020*	0.403	0.162
lnvaspin (NGT)					
Model 1: ln(RFa deep breathing)	(−3.16, p = 0.002)	10.0 (1.68)	*p = 0.002*	0.358	0.128
lnvaspin (NGT – MetS)					
Model 1: ln(RFa deep breathing)	(−2.99, p = 0.005)	8.92 (1.33)	*p = 0.005*	0.461	0.213
lnvaspin (NGT + MetS)					
Model 1: ln(LFa at rest)	(−2.31, p = 0.027)	5.36 (1.33)	*p = 0.027*	0.374	0.140

ln—natural logarithmic transformation for skewed data distribution

Statistical significant values are presented in italics

## Discussion

Our results demonstrated no significant difference in vaspin levels between the groups according to glucose tolerance and the presence of MetS. No correlation between serum vaspin and IRI levels, HOMA-IR and HOMA-β was established in the same groups as well. In accordance with our results some recent studies have presented no difference in vaspin levels between subjects with T2D and MetS as compared to those without MetS [9], or even lower vaspin concentrations in men with MetS [10] and a reciprocal relation between serum vaspin and insulin levels [11]. Other studies have found no difference in vaspin levels between NGT women with and without obesity [12] and reported no relationship between vaspin concentrations and insulin sensitivity in men [13] and in both genders [14] as well.

Despite some observations for an independent relation between serum vaspin and VAT in the presence of high HOMA-IR [15], our results revealed a significant correlation between serum vaspin and total body fat in NDT2D and in the presence of MetS in accordance with Kloting et al. study. This controversy is probably due to the fact that vaspin mRNA expression have been described just in 23 % of VAT and 15 % of subcutaneous adipose tissue (SAT) samples and no correlation between visceral vaspin gene expression and VAT and SAT areas have been observed [16]. Furthermore, another study has reported vaspin mRNA expression predominantly in nonfat cells [17].

A number of previously reported data have shown a correlation between vaspin levels and insulin sensitivity and obesity indices [15, 18–22]. Flehming et al. have conducted cluster analysis of 20 adipokines, including vaspin, to compare their predictive value for the presence of T2D with a set of traditional markers—HbA1c, HOMA-IR and fasting plasma glucose. Contrary to the expectations this cluster has shown lower sensitivity and specificity [23]. Hence, although it is assumed that vaspin has putative insulin-sensitizing properties, probably the relationship between vaspin and the parameters of insulin sensitivity might be significantly altered by the presence of glucose tolerance impairments in obese individuals.

We found a positive correlation between serum vaspin and AGEs accumulation in subjects with MetS independently of their glucose tolerance. It has been implied that vaspin protects endothelial cells via inhibition of NF-kB [24]. On the other hand, endothelial injury is mediated by AGEs via up-regulation of the same transcription factor [25]. In this line it could be assumed that vaspin serves as a compensatory mechanism against to oxidative stress in MetS.

It has been suggested that vaspin exerts anti-inflammatory effects [22] and hsCRP independently predicts circulating vaspin level in chronic dialysis patients [26]. Contrary to the above, we did not identify any correlation between vaspin and hsCRP in the studied groups. A limitation of our analyses is that inflammation has been only characterized by circulating hsCRP and inclusion of additional markers of inflammation may change this result.

Kempf et al. have identified a correlation between vaspin gene single nucleotide polymorphism rs2236242 and T2D with genotype AA. It has been assumed that vaspin might be a new link between obesity and related glucose metabolism disorders [27]. Our findings revealed a negative correlation between serum vaspin and fasting plasma glucose and HbA1c levels in NDT2D group, whereas there was no correlation between vaspin and glycemic parameters in prediabetes and NGT groups. Although we observed a trend towards higher serum vaspin levels in the groups with the worsening of glucose intolerance, the difference was not statistically significant. Li et al. have reported similar results, establishing elevated serum vaspin only in NDT2D [28]. Other authors have observed elevated vaspin levels in obese subjects with NGT [29] and in prediabetes [30]. Data on serum vaspin levels in T2D are rather conflicting. Some studies have demonstrated increased vaspin concentrations [28, 31], others have reported no difference in vaspin levels [18], or even decreased vaspin concentrations [32, 33] in T2D. Taking into account diabetes duration Atya et al. and Feng et al. have found a reduction in circulating vaspin in subjects with longer diabetes duration [29, 34]. Based on these ambivalent data it could be speculated that, if vaspin plays a compensatory role in glucose metabolism disorders, it manifests at relatively early stages of glucose intolerance, namely at the onset of T2D, and its secretion capacity gradually declines with the increase of diabetes duration.

There are limited data on vaspin relation to diabetes chronic complications. Gulcelik et al. have demonstrated diminished vaspin levels in the presence of microvascular complications [32] and Li et al. in the presence of macroangiopathy [35]. It has been suggested that vaspin regulates eNOS function in endothelial progenitor cells in subjects with diabetes and thus prevents the occurrence of vascular complications [36]. If there is any impact on peripheral nerve fibers is still unclear. As far as we know the relation between vaspin levels and CAF has been investigated only in adolescent subjects with type 1 diabetes and there are no data in adults. El Dayem et al. study has shown a significant relation between serum vaspin and a standard deviation difference RR as a time domain HRV parameter of CAF, assessed by 24-h holter monitoring [37]. Our findings displayed a significant negative correlation between vaspin and both sympathetic and parasympathetic activity at rest and during clinical tests just in NGT group. After subdividing this group according to the presence of MetS serum vaspin was independently related to sympathetic activity in NGT + MetS group and to parasympathetic activity in NGT – MetS group. As there was no association between vaspin concentrations and autonomic activity in the groups with glucose intolerance, it has probably been obscured by dysglycemia. Vinik’s classification of CAD stages based on the high-sensitive ANX-3.0 method, applied in the present study, defines early parasympathetic weakness [38], which is observed even in the absence of insulin resistance [39]. This assumption clarifies the negative correlation of vaspin with parasympathetic tone in NGT – MetS group and with sympathetic tone only in the presence of MetS. Insulin resistance, endothelial dysfunction and overproduction of adipokines are likely accompanying mechanisms for the development of CAD [40, 41]. Based on our finding and Chang et al. data [39] it might be speculated that probably insulin resistance is a consequence of existing autonomic damage and vaspin exerts its protective effects long before the development of metabolic syndrome abnormalities.

## Conclusion

Our results demonstrate no overt fluctuations in vaspin levels in the early stages of glucose intolerance and in MetS. Total body fat and age seem to influence vaspin in MetS, and just total body fat in NDT2D. Our data show negative correlation between vaspin and CAF in normoglycemia, as vaspin is associated with parasympathetic activity even in the absence of MetS.

## Abbreviations

MetS: metabolic syndrome
NGT: normal glucose tolerance
T2D: type 2 diabetes,
NDT2D: newly-diagnosed type 2 diabetes
BMI: body mass index
SAT: subcutaneous adipose tissue
VAT: visceral adipose tissue
IRI: immunoreactive insulin
HOMA-IR: homeostasis model assessment of insulin resistance
HOMA-β: homeostasis model assessment of -beta-cell function
hsCRP: high sensitive C-reactive protein
IL-6: interleukin-6
GLUT-4: glucose transporter-4
AGEs: advanced glycation end products
CAF: cardio-vascular autonomic function
CAD: cardio-vascular autonomic dysfunction
HRV: heart rate variability
RFa: respiratory frequency area
LFa: low frequency area
mRNA: messenger ribonucleotide acid
IDF: International Diabetes Federation
WHO: World Health Organization
